# AI-assisted assessment of the IFSO consensus on obesity management medications in the context of metabolic bariatric surgery

**DOI:** 10.1371/journal.pdig.0001132

**Published:** 2025-12-19

**Authors:** Mohammad Kermansaravi, Paulina Salminen, Gerhard Prager, Ricardo V. Cohen

**Affiliations:** 1 Division of Minimally Invasive and Bariatric Surgery, Department of Surgery, Minimally Invasive Surgery Research Center, Hazrat-E Fatemeh Hospital, Iran University of Medical Sciences, Tehran, Iran; 2 Division of Digestive Surgery and Urology, Turku University Hospital, Turku, Finland; 3 Department of Surgery, University of Turku, Turku, Finland; 4 Division of Visceral Surgery, Department of General Surgery, Vienna Medical University, Vienna, Austria; 5 The Center for the Treatment of Obesity and Diabetes, Hospital Alemao Oswaldo Cruz, Sao Paulo, Brazil; Tohoku Daigaku - Seiryo Campus, JAPAN

## Abstract

Artificial intelligence (AI) and large language models (LLMs), when combined with human expertise in collaborative intelligence (CI), can enhance medical decision-making, reduce bias in guideline development, and support precision care. New obesity management medications (OMMs) such as GLP-1 receptor agonists and dual incretin mimetics complement metabolic bariatric surgery but currently lack clear integration strategies. To address this gap, IFSO released consensus guidelines in 2024. This study evaluates their robustness by comparing expert recommendations with LLM outputs, highlighting the role of AI in assessment and strengthening clinical consensus. Thirty-one IFSO consensus statements were tested across eleven advanced LLMs on June 1, 2025. Models received standardized prompts that required binary “AGREE” or “DISAGREE” outputs, supported by brief, evidence-based rationales. Individual responses were aggregated to form an overall “LLM consensus,” and mean percentage agreement was calculated against the original IFSO expert grades—Fleiss’ κappa quantified inter-model reliability beyond chance. Incorporating the AI responses led to shifts in the consensus grade for 2 of the 31 statements. One statement originally rated A + was downgraded to A after some LLMs’ outputs indicated disagreement, citing nuanced evidence on pre- and post-MBS OMM use and comparative effectiveness. One statement on combining OMMs with endoscopic therapies was upgraded from C to B due to unanimous support from the LLM. The remaining 29 statements maintained their original grades, demonstrating strong overall alignment between LLM outputs and expert consensus. Overall concordance between LLMs and experts was 93%, with substantial inter-model agreement(κ = 0.81 [95% CI 0.74–0.87]). Integrating AI, especially LLMs, into collaborative intelligence frameworks strengthens clinical consensus when evidence is limited. This study shows that concordance between LLMs outputs and expert consensus should not be taken as evidence of objectivity; rather, it may simply reflect overlap between the published evidence base and the model’s training data or retrieval sources.

## Author summary

In this study, we explored how artificial intelligence (AI), specifically large language models (LLMs), can work with human experts to support medical decision-making. We focused on new obesity management medications, which are emerging quickly but still lack clear guidance on how they should be combined with metabolic and bariatric surgery. In 2024, the International Federation for the Surgery and Other Therapies of Obesity (IFSO), released consensus guidelines, and we set out to examine the strength and consistency of those recommendations. We asked eleven advanced LLMs to evaluate thirty-one statements from the guidelines. Each model indicated whether it agreed or disagreed with the statements and provided short explanations. We compared these responses with the original expert recommendations. Most statements showed strong alignment between models and experts. Only two statements changed in strength: one was downgraded because some models pointed out limited supporting evidence, and another was upgraded because all models endorsed it. Overall, agreement was high. Our findings suggest that AI can help assess clinical consensus when evidence is evolving. At the same time, close alignment between LLMs and experts should not be mistaken for greater objectivity, as the models may simply reflect the evidence they were trained on.

## Introduction

The development of artificial intelligence (AI) and large language models (LLMs) has commenced a new era in medicine and surgery, offering scalable and evidence-informed support for healthcare professionals, students, and patients. AI and machine learning are revolutionizing healthcare and biomedical research, with proven benefits in data extraction, diagnosis, personalized care, and clinical decision support. Continued progress depends on addressing challenges in data, interpretability, and regulation to ensure the responsible and impactful integration of AI into clinical practice [1,2]. AI now sits at the core of contemporary care, powering high-accuracy diagnosis and protecting patient confidentiality. Widely adopted approaches, deep learning for clinical insight, federated learning for decentralized model training, and privacy-preserving machine learning for secure computation, each address distinct clinical, operational, and ethical requirements [3,4]. AI improves diagnostic accuracy and efficiency while embedding safeguards that protect sensitive patient data, expanding what medical AI can responsibly achieve [4]. By learning from large, diverse datasets, these systems flag risk earlier, anticipate disease trajectories, and guide timely, personalized treatment plans that better fit each patient [5]. When combined with human expertise in a collaborative intelligence (CI) framework, AI can enhance decision-making by uniting computational speed with ethical and clinical reasoning [6]. Integrating CI into expert consensus development - whether in Delphi rounds or post-hoc assessment - offers a promising approach to improving the transparency and objectivity of clinical guidelines. By synthesizing vast volumes of medical literature, LLMs provide timely, evidence-based input that may support balanced expert deliberation, helping to reduce common biases such as groupthink or dominance effects [7–9]. This synergy between human insight and AI capabilities aligns with the priorities of modern healthcare, where individualized, data-informed care is critical. CI facilitates the standardization of practice while enabling adaptive, precision-guided decisions [10,11].

In this evolving obesity treatment landscape, obesity management medications (OMMs) have emerged as valuable tools to complement metabolic bariatric surgery (MBS). These agents—particularly GLP-1 receptor agonists and dual incretin mimetics—have demonstrated significant weight loss and metabolic benefits, serving both as alternatives for non-surgical candidates and as adjuncts to improve surgical outcomes [12,13]. However, evidence on the optimal integration of OMMs with MBS remains limited, and clear strategies for their integration are lacking. To address this, the International Federation for the Surgery and Other Therapies of Obesity (IFSO) recently published a consensus statement offering clinical guidance on the use of OMMs in the pre-, peri-, and postoperative phases of MBS [14].

This study aims to assess the alignment between this evidence-based expert consensus and AI-generated outputs using a collaborative intelligence approach.

## Methods

To evaluate the alignment between AI (LLMs) and expert clinical consensus, we input all 31 statements from the IFSO International Consensus Position Statement [14] into 11 advanced LLMs on the same day (June 1, 2025). The LLMs used in this study included: ChatGPT-4 o, Gemini 2.5 Pro, BioGPT, PubMedGPT, DeepSeek, Grok 3, MedGPT, Gemma 3n E4B, Qwen2.5-Max, Microsoft Copilot, and Claude 3.7 Sonnet. The 11models were chosen because they were publicly accessible on June 1, 2025, and represent the majority of real-world clinical use. We acknowledge that several models share architecture families and/or overlapping training corpora (e.g., Copilot and GPT-4 lineage models; PubMedGPT vs BioGPT for biomedical corpora). For this reason, we treat “LLM consensus” not as 11 independent statistical samples but as a *model ecosystem signal* for qualitative convergence. Advanced non-public research models were excluded due to access restrictions.

For transparency and reproducibility, we report model version identifiers, access date, last known training data cutoff, domain specialization, and whether real-time retrieval was available in S1 Table.

Prompt standardization was ensured by using a single, uniform prompt format: each IFSO statement was presented to every model with instructions to answer “AGREE” or “DISAGREE” and to provide a brief rationale grounded in current clinical evidence or guidelines; the exact, unmodified following prompt was applied identically to each statement for all models.

***“You are an evidence-based clinical decision support system. Evaluate the following IFSO consensus statement. Respond using ONLY one of two options: AGREE or DISAGREE. Provide a concise evidence-based justification relying solely on established peer-reviewed data or clinical guidelines. Do not speculate.”***

To minimize potential hallucinations, models were explicitly directed to avoid speculation and to base responses only on established medical knowledge. To mitigate the risk of the subjective influence, all LLM outputs were collected independently, using a pre-specified prompt structure, on a single predefined date, and without manual editing or reinterpretation of the models’ responses. Agreement grading was algorithmically derived from raw “agree/disagree” outputs, not from subjective narrative interpretation. Two independent coders (M.K. and R.V.C.) independently classified each response strictly by the first token; if the model began with “AGREE” it was coded AGREE, if it began with “DISAGREE” it was coded DISAGREE, regardless of qualifiers. Ambivalent statements (e.g., conditional language) were not re-interpreted by the coders. Inter-coder reliability for the 31 × 12 = 372 classifications was 100% because the first token rule is unambiguous and deterministic. All responses were collected and analyzed to generate a consolidated “LLM Consensus.” In the final phase, the mean percentage agreement between the IFSO expert consensus and the aggregated LLM consensus was calculated. This means the percentage served as the basis for determining the overall outcome of what we define as the “Collaborative Intelligence Consensus”.

To categorize the level of consensus support, we adopted the same grading system used in the IFSO consensus study (Table 1). Using this standardized classification simplified our evaluation and allowed for direct comparison with the IFSO consensus findings, allowing us to assess both the degree of concordance between LLMs and human experts and to evaluate the potential of CI as a robust method for post-hoc assessment of clinical consensus statements. In addition to mean percentage agreement, we calculated *Fleiss’* κ*appa* to quantify inter-model agreement beyond chance across all 31 statements and 11 LLMs. Each statement was coded as “AGREE” or “DISAGREE,” and κappa was derived from the aggregated 31 × 11 binary matrix using standard methods for multiple raters. Interpretation followed the Landis–Koch benchmarks, where 0.61–0.80 represents substantial and >0.80 almost-perfect agreement.

**Table 1:** The Delphi expert percentage consensus support for each statement.

Consensus Support	Definition
**Grade A+**	• 100% agreement across all LLMs and the expert panel
**Grade A**	• 90–99.9% agreement
**Grade B**	• 80–89.9% agreement
**Grade C**	• 70–79.9% agreement
**Grade D**	• 66–69.9% agreement
**Failed Consensus**	• Less than 66% agreement

## Results

Incorporating the AI responses led to shifts in the consensus grade for 2of the 31 statements (Statements 4 and 16; see Table 2). One statement originally graded A+ by the IFSO expert consensus was downgraded to A based on the aggregated LLM responses, whereas one statement graded C by the experts was upgraded to B in the LLM-aggregated assessment.

**Table 2:** AI-augmented consensus on obesity medications in metabolic bariatric surgery.

Statement	Expert Consensus	LLMs Consensus	Collaborative Intelligence Consensus
1. Clinical obesity is a disease that requires treatment	A+ (100%)	A+ (100%)	A+ (100%)
2. Patients should be informed of the risks and benefits of evidence-based treatment options for obesity	A+ (100%)	A+ (100%)	A+ (100%)
3. A minimum of 5% weight loss has shown metabolic improvements; however, greater weight loss is associated with broader clinical benefits, including a reduction in mortality	A (97%)	A+ (100%)	A (98%)
4. There is insufficient high-level evidence to recommend the routine use of OMMs for weight loss before MBS	A+ (100%)	B (82%)	A (91%)
5. The decision to use OMMs before MBS should be personalized to determine the most appropriate strategy for each patient’s circumstances	A+ (100%)	A+ (100%)	A+ (100%)
6. Future research is needed to explore the value of using OMMs before MBS to assess their benefits, risks, and clinical outcomes	A+ (100%)	A+ (100%)	A+ (100%)
7. Healthy nutrition, including adequate protein consumption, as well as resistance exercise, is recommended for those treated with OMMs before MBS	A (97%)	A+ (100%)	A (98%)
8. In general, preoperative treatment with OMMs should be discontinued before MBS to minimize perioperative risk	A (94%)	A (92%)	A (93%)
9. Treatments with OMMs after MBS should generally be withheld until the achievement of the weight plateau, unless there is a compelling clinical need for earlier initiation	A+ (100%)	A+ (100%)	A+(100%)
10. Future research is needed to identify predictors of which patients are likely to derive substantial benefit from combined pharmaco-surgical therapy for obesity and its complications	A+ (100%)	A+ (100%)	A+ (100%)
11. MBS is strongly associated with reduced adverse cardiovascular events, and GLP-1 receptor agonists (GLP-1 RAs) have been shown to reduce such events. Future research is required to determine the benefits of combination treatment for these outcomes.	A+ (100%)	A+ (100%)	A+ (100%)
12. Both MBS and GLP-1 RAs agonists reduce chronic kidney disease. Future research is required to determine the benefits of combination treatment for these outcomes.	A+ (100%)	A+ (100%)	A+ (100%)
13. In patients with a suboptimal clinical response after MBS, the addition of OMMs can improve metabolic outcomes	A+ (100%)	A+ (100%)	A+ (100%)
14. For patients requiring OMMs to maintain a healthy weight after MBS, the ongoing use of the medications is likely needed	A (94%)	A+ (100%)	A (97%)
15. Research on the intermittent use of OMMs and/or their dose adjustment after MBS with a suboptimal response is needed	A (94%)	A+ (100%)	A (97%)
16. The benefit of endoscopic therapies for obesity can be enhanced by the combination with OMMs	C (74%)	A+ (100%)	B (87%)
17. Patients with a suboptimal initial response or recurrent weight gain after MBS should be informed of all available evidence-based treatments, including their benefits and risks	A+ (100%)	A+ (100%)	A+ (100%)
18. In patients with a suboptimal initial response or recurrent weight gain after MBS, different options, including OMMs, endoscopic therapies, and revisional and conversion surgery, can be considered	A (94%)	A+ (100%)	A (97%)
19. Emerging evidence indicates that the weight loss induced by OMMs is similar among people who have or have not undergone MBS	A+ (100%)	A+ (100%)	A+ (100%)
20. When used after MBS, there appears to be no increased incidence of side effects of OMMs compared to non-surgical cohorts	A (97%)	A+ (100%)	A (98%)
21. As the long-term efficacy and safety of OMMs after MBS is unknown, studies are needed to understand the value and limitations of such combination therapy	A+ (100%)	A+ (100%)	A+ (100%)
22. Endpoints of future clinical trials of existing and/or novel obesity-management interventions (behavioral, pharmacological, endoscopic, and surgical) should focus on improvement, remission, and prevention of clinical manifestations and complications of obesity in addition to weight loss	A+ (100%)	A+ (100%)	A+ (100%)
23. Studies are needed to define stage-specific therapeutic protocols that integrate surgical intervention and adjuvant pharmacotherapy to achieve improvement (or remission when possible) of clinical obesity	A (95%)	A+ (100%)	A (97%)
24. Further investigation of the mechanisms of action of distinct MBS procedures is an important research priority to understand the additive vs. synergistic effects of different possible combinations of surgical and drug-based therapies. This knowledge is necessary to optimize the safety and efficacy of adjuvant pharmacotherapy for obesity.	A (95%)	A+ (100%)	A (97%)
25. For patients with recurrent weight gain, treatment with available OMMs should be considered prior to revisional surgery.	A (92%)	A+ (100%)	A (96%)
26. If treatment with OMMs after MBS results in a suboptimal clinical response or if there is an inability to continue medications (e.g., due to cost or an adverse reaction), then endoscopic, revision, or conversion surgery should be considered.	A+ (100%)	A+ (100%)	A+ (100%)
27. People living with obesity need access to all evidence-based treatments, including MBS and OMMs, as part of standard healthcare services	A (95%)	A+ (100%)	A (97%)
28. Health systems need to support the long-term management of obesity as they do for other chronic diseases (e.g., diabetes or cardiovascular disease)	A (95%)	A+ (100%)	A (97%)
29. All healthcare providers need a basic understanding of the complex etiology, pathophysiology, and evidence-based management of obesity	A+ (100%)	A+ (100%)	A+ (100%)
30. Studies on the cost-effectiveness of the association of modern pharmacotherapy and MBS are essential to determine the role of preoperative and postoperative OMMs	A+ (100%)	A+ (100%)	A+ (100%)
31. Similar benefit-risk and benefit-cost considerations, and therefore willingness to pay, should be applied to the treatment of obesity as they are to other chronic diseases	A+ (100%)	A+ (100%)	A+ (100%)

Although most of the included LLMs did not provide references for their responses, none relied on unrealistic or incorrect inferences, and in the majority of cases, they appropriately acknowledged the presence or absence of supporting clinical evidence.

Statement 4, “*There is insufficient high-level evidence to recommend the routine use of OMMs for weight loss before MBS,”* subject to full consensus in the IFSO position statement, received broad agreement from most LLMs; two models, *Qwen2.5-Max and Claude 3.7 Sonnet outputs, indicated disagreement*. As a result, the level of agreement was downgraded from A+ to A. *Qwen2.5-Max responded: “DISAGREE – While high-level evidence may be limited, some oral medications for weight management (OMMs) have shown efficacy in specific populations.* However, their routine use before MBS should still be individualized.” *Claude 3.7 Sonnet similarly disagreed, stating: “Growing evidence supports preoperative OMM use in selected patients for improved surgical outcomes.”*

Conversely, the statement 16, “*The benefit of endoscopic therapies for obesity can be enhanced by the combination with OMMs*”, was upgraded from Grade C to Grade B within the aggregated LLM responses. We reviewed the rationales produced by the LLMs to identify the underlying evidence they cited. Most LLM rationales referenced small, recent pilot studies, case series, and narrative reviews suggesting potential additive benefit when endoscopic procedures (for example, endoscopic sleeve gastroplasty) are combined with pharmacotherapy (notably GLP-1 receptor agonists). However, the literature identified by the models is limited in size, heterogeneity, and follow-up duration, and high-quality randomized controlled trials (RCTs) demonstrating clear additive or synergistic effects are not yet available [15].

The remaining 29 statements retained their original level of recommendation, indicating a high degree of alignment between the expert consensus and the LLM-derived responses across most areas (Table 2). The complete results are provided in S2 Table. Beyond raw percentage concordance, overall inter-model reliability was high (Fleiss’ κ = 0.81 [95% CI 0.74–0.87]), indicating substantial agreement among the 11 LLMs beyond chance. This quantitative metric corroborates the qualitative alignment observed between LLM outputs and expert consensus.

## Discussion

The field of metabolic bariatric surgery (MBS) is currently experiencing two major breakthroughs. First, the emergence of a new generation of OMMs offers promising potential to complement and enhance the outcomes of MBS procedures. These medications can serve as valuable adjuncts, reinforcing the effects of MBS and supporting long-term weight management [12]. Second, the integration of artificial intelligence (AI) into medicine, including the field of MBS, is opening new frontiers. AI is proving its value across healthcare, helping catch problems earlier, streamlining busywork, and tailoring care to each person. As these tools spread from clinics to back offices and research labs, care becomes more accurate, efficient, and personal, opening the door to better access and outcomes for more patients [1]. AI in healthcare safeguards patient data through advanced de-identification and strong encryption or differential privacy with encryption in transit and at rest [16], while simultaneously optimizing hospital logistics across supply chains, staffing, patient flow, and operating rooms to improve operational efficiency and care quality [17].

By combining AI, including LLMS, with human clinical expertise and evidence-based practice, a form of collaborative intelligence (CI) is emerging that enhances medical decision-making, reduces bias in guideline development, and supports more precise, patient-specific care [6]. When paired with blockchain for secure, transparent, and efficient handling of medical data, these complementary technologies can further improve decision quality and ultimately strengthen patient outcomes [18,19]. This synergy can significantly improve decision-making, particularly in critical situations or when clear, high-level evidence and established guidelines are lacking [6]. In such cases, AI can help synthesize existing data to guide consensus-driven decisions grounded in the best available evidence. In addition, CI marks a significant advancement and next-generation evolution of the traditional Delphi method, reimagined to address the increasing complexity, speed, and uncertainty of today’s decision-making landscapes. It facilitates a dynamic, flexible, and ongoing consensus process that harnesses the distributed expertise of diverse contributors, enhanced and coordinated through AI-driven coordination. LLMs can act as synthetic panel members to generate, evaluate, and even augment consensus statements in group decision-making and evidence appraisal. When used to augment consensus or policy preference data, LLMs can enhance the accuracy of aggregate estimates beyond what traditional, non-augmented samples can achieve. When guided by human oversight, they can meaningfully augment the interpretation and outcomes of surveys or consensus-building efforts [20].

Similar to the IFSO Consensus, the LLMs reached agreement on all 31 statements, providing further evidence that the IFSO consensus was well grounded in the existing body of evidence. This alignment demonstrates that the IFSO recommendations were not influenced by subjective opinion alone but were firmly supported by the available data. The LLM outputs indicated potential changes in the strength of recommendation for only 2 of the 31 statements (Statements 4and 16): one was downgraded slightly from A+ to A, and one was adjusted from grade C to grade B. These modifications were minimal, and none of the AI feedback materially changed the overall consensus conclusions. In statement 4 (There is insufficient high-level evidence to recommend the routine use of OMMs for weight loss before MBS), where models disagreed with the IFSO consensus, their outputs indicated alternative interpretations of the existing evidence, often emphasizing conditional or patient-specific considerations. These outputs reflect statistical patterns learned from training data and retrieval behavior rather than intentional clinical judgment. We therefore describe model responses as indications of alternative evidence syntheses or emphases rather than as demonstrations of clinical reasoning.

Regarding statement 16 (The benefit of endoscopic therapies for obesity can be enhanced by the combination with OMMs), the consensus among AI models likely reflects their access to the latest publications and preprints, enabling them to detect and synthesize patterns of evidence not yet fully consolidated in expert guidelines. Given the predominance of low-to-moderate quality evidence, the original expert panel’s C rating (reflecting limited or emerging evidence) remains defensible. We therefore interpret the LLM upgrade as an early-signal detection of heterogeneous, low-certainty evidence rather than as definitive confirmation of a robust, high-quality evidence base [15]. This distinction is important when considering whether model agreement should prompt changes to guideline recommendations. This finding illustrates how the CI approach can complement expert opinion by surfacing early signals of evolving clinical practice, thereby offering a more responsive and adaptive framework for evidence appraisal.

It is important to note that, unlike human expert consensus, LLMs do not engage in deliberative discussions and consensus-building influenced by expert dialogue. Instead, it relies strictly on available data and evidence. While AI, defined as autonomous computational systems that learn from raw data, has not yet achieved the depth of clinical reasoning, contextual awareness, and experiential insight characteristic of healthcare professionals [21], its alignment with the IFSO Consensus lends strong support to expert-driven recommendations. It is incorrect to assume that LLMs are free from human bias. LLM outputs are influenced by the data and curation choices used during model training and by retrieval sources; they can therefore reflect, amplify, or re-weight biases present in published literature or online material. We have revised the manuscript to explicitly acknowledge algorithmic bias as a major limitation of AI-augmented evidence appraisal. Notable discussions of these risks include Bender et al. [22] and Weidinger et al. [23], which highlight the potential for LLMs to reproduce and amplify problematic patterns from their training corpora. The current study shows AI can synthesize large literatures and reduce bias in guideline workflows; the strong concordance between expert and model judgments across 29/31 statements mirrors those findings by indicating AI’s value as a decision-support adjunct rather than a replacement for expert panels.

While the present study evaluated the IFSO consensus after publication, a future CI workflow can be implemented *during* guideline development. A prospective CI-enhanced consensus process would occur in real time and would be structured in parallel with Delphi rounds. In each round, the expert panel would generate suggested statements, and the same statements would simultaneously be submitted to pre-specified LLMs using a locked prompt, locked model versions, and locked access dates. LLM outputs would be tabulated (“agree/disagree + evidence strings”) and integrated into the Delphi matrix, not as votes, but as external evidence signals. The expert panel would then review (but not automatically adopt) LLM-derived patterns before proceeding to the next round.

Prospective implementation would also require explicit governance for disagreement. If one or more LLMs yield a conditional or divergent interpretation relative to the expert majority, the panel would document the discordance and evaluate the underlying references using formal quality appraisal tools. This would allow LLMs to surface potentially overlooked literature while maintaining human adjudication of evidentiary strength. Importantly, LLM access and cutoffs would be locked per round so that models cannot access temporally newer evidence than experts (temporal bias). AI-augmented CI, therefore, becomes a procedural element of Delphi, rather than a post-hoc commentary. A recently published paper using LLM-mediated Delphi procedures found that LLM-derived consensus tracked closely with human expert judgments, while the models’ simulated deliberation remained largely guideline-oriented and predisposed toward conservative positions [24].

It seems entirely reasonable that in the future, guidelines, position statements, and Delphi processes, and their updates, will be developed with the support of collaborative intelligence.

## Future outlook

Looking ahead, collaborative intelligence could be formally integrated into regulatory and Delphi frameworks as a procedural step in evidence synthesis and guideline development. By establishing governance standards for model selection, prompt transparency, and provenance tracking, CI could evolve from a proof-of-concept tool to a recognized methodological layer within consensus processes. This approach would allow regulatory bodies, professional societies, and expert panels to incorporate AI-augmented evidence review in real time, ensuring that future recommendations remain both data-responsive and ethically grounded.

## Limitations and strengths

The first limitation is that this AI-assisted assessment study was conducted following the publication of the IFSO Consensus. While none of the LLMs directly cited or explicitly referenced the consensus document in their outputs, elements of the consensus were probably incorporated into the models’ training data, given its public availability. Given the opaque nature of LLM training corpora, there is also a potential risk of data contamination, and the models may have been trained, in whole or in part, on the IFSO Consensus itself or related materials, although none of the LLMs explicitly referenced or cited the IFSO Consensus document. This possibility should be considered when interpreting the models’ performance and alignment with expert-derived standards, as it may influence their apparent agreement with consensus findings. To minimize this potential overlap and bias in future research, similar studies should be conducted in parallel with the experts’ consensus-building process and before the publication of final consensus statements and results, to ensure the independence and objectivity of AI-generated insights. Another limitation was the restricted access to several specialized medical LLMs developed by leading academic institutions around the world. Due to their proprietary nature, these models could not be included in the analysis, which may have limited the breadth of perspectives captured.

In addition, the authors of this study were members of the expert panel that authored the IFSO consensus being evaluated. This dual role presents a risk of confirmation bias, since authors may unintentionally favor interpretations that preserve or validate previously published recommendations. This potential influence should be acknowledged when interpreting the results.

The strengths of this study included the employment of the publicly accessible versions of the 11 most prominent and widely used LLMs. To minimize variability and ensure methodological consistency, all models were queried individually but on the same day, using an identical set of prompts. This simultaneous data collection approach mitigated the potential for temporal bias and controlled for the influence of newly published literature or updates that could have skewed results. This underscores a broader challenge in evaluating LLM performance when expert benchmarks are publicly accessible, i.e., distinguishing genuine inferential reasoning from outputs that may reflect memorized or reproduced content. Despite the authors’ overlap with the IFSO consensus, an AI concordance assessment remains informative because the evaluation uses LLM-generated outputs produced without exposure to the authors’ reasoning process, and because the grading system is directly computed from binary model decisions rather than from human interpretation. Importantly, disagreements were not suppressed; they are explicitly reported, and in two cases resulted in downgrading the expert consensus. This transparency strengthens the credibility of the findings and demonstrates that collaborative intelligence can challenge, rather than rubber-stamp, expert opinion. While the high concordance between aggregated LLM outputs and the IFSO expert panel across the majority of statements is notable, concordance alone does not constitute independent validation. The substantial κappa value (0.81) further supports that the observed alignment reflects genuine inter-model consistency rather than random or coincidental agreement, reinforcing the robustness of the collaborative-intelligence approach. High agreement can reflect shared exposure to the same literature, LLM memorization of widely available guidance, or retrieval of similar sources. Future studies should focus on systematic evaluation pipelines that combine LLM outputs with independent critical appraisal and prospectively withheld validation corpora.

## Conclusion

The integration of artificial intelligence, particularly large language models, into clinical decision-making frameworks can be a valuable strategy when high-level evidence is limited and expert consensus becomes essential. Collaborative intelligence can be a useful adjunct to highlight convergent interpretations, surface recent or under-appreciated literature, and identify statements that deserve closer evidentiary scrutiny. However, CI should not be used as a stand-alone validator of expert guidance without careful provenance tracking, quality appraisal of underlying evidence, and independent external review.

## Supporting information

S1 Table Model metadata (public production versions accessed on 1 July 2025). (DOCX)

S2 Table AI-augmented consensus on obesity medications in metabolic bariatric surgery. (DOCX)
