# Supplementary material for: AI-assisted assessment of the IFSO consensus on obesity management medications in the context of metabolic bariatric surgery
Source: PLOS Digit Health. 2025 Dec 19;4(12):e0001132. doi: 10.1371/journal.pdig.0001132 (PMC12716726; doi:10.1371/journal.pdig.0001132)
Supplement: S1 Table — (DOCX) [file pdig.0001132.s001.docx]

# S1 Table. Model metadata (public production versions accessed on 1 July 2025)

| Model (UI label at time of use) | Version identifier / build label (publicly stated or manufacturer-equivalent) | Approx. knowledge cutoff (published / model-card) | Domain specialization | Real-time retrieval |
| --- | --- | --- | --- | --- |
| ChatGPT-4o (OpenAI) | gpt-4o-2024-05-13 | ~2023-late (multi-year composite) | General-purpose | No |
| Gemini 2.5 Pro (Google DeepMind) | Gemini-2.5-Pro-2025-05 | ~2024 | General-purpose | No |
| Claude 3.7 Sonnet (Anthropic) | claude-3.7-sonnet-2025-05-alpha | ~early-2024 | General-purpose | No |
| BioGPT (Microsoft Research) | BioGPT-base-v1.5 | PubMed up to ~2022 | Biomedical | No |
| PubMedGPT (Stanford) | PubMedGPT-clinical-2023 | PubMed ~2023 | Biomedical | No |
| DeepSeek (DeepSeek) | DeepSeek-V3-2025-05 | ~2023–2024 | General | No |
| Grok 3 (xAI) | Grok-3-2025-Q2 | ~2024 | General | No |
| MedGPT (OpenMedicalAI) | MedGPT-2024-Q4 | ~2023/2024 (biomed heavy) | Biomedical | No |
| Gemma 3n E4B (Google) | Gemma-3n-E4B-2025-alpha | ~2024 | General | No |
| Qwen2.5-Max (Alibaba) | Qwen-2.5-Max-2025-05 | ~2024 | General | No |
| Microsoft Copilot | ChatGPT-4-family derivative (gpt-4o-2024-05-13 basis) | ~2023 | General | No |
