# Supplementary material for: AI-assisted assessment of the IFSO consensus on obesity management medications in the context of metabolic bariatric surgery
Source: PLOS Digit Health. 2025 Dec 19;4(12):e0001132. doi: 10.1371/journal.pdig.0001132 (PMC12716726; doi:10.1371/journal.pdig.0001132)
Supplement: S2 Table — (DOCX) [file pdig.0001132.s002.docx]

**S2 Table - AI-Augmented Consensus on Obesity Medications in Metabolic Bariatric Surgery**

| **Statement** | **Expert**  **Consensus** | **ChatGPT 4 o** | **Gemini 2.5 pro** | **BioGPT** | **PubMedGPT** | **DeepSeek** | **Grok 3** | **MedGPT** | **Gemma 3n E4B** | **Qwen2.5-Max** | **Copilot** | **Claude3.7 Sonnet** | **LLMs Consensus** | **Collaborative Intelligence Consensus** |
| --- | --- | --- | --- | --- | --- | --- | --- | --- | --- | --- | --- | --- | --- | --- |
| 1. **Clinical obesity is a disease that requires treatment** | **A+ (100%)** | **Agree** | **Agree** | **Agree** | **Agree** | **Agree** | **Agree** | **Agree** | **Agree** | **Agree** | **Agree** | **Agree** | **A+ (100%)** | **A+ (100%)** |
| 1. **Patients should be informed of the risks and benefits of evidence-based treatment options for obesity** | **A+ (100%)** | **Agree** | **Agree** | **Agree** | **Agree** | **Agree** | **Agree** | **Agree** | **Agree** | **Agree** | **Agree** | **Agree** | **A+ (100%)** | **A+ (100%)** |
| 1. **A minimum of 5% weight loss has shown metabolic improvements; however, greater weight loss is associated with broader clinical benefits, including a reduction in mortality** | **A (97%)** | **Agree** | **Agree** | **Agree** | **Agree** | **Agree** | **Agree** | **Agree** | **Agree** | **Agree** | **Agree** | **Agree** | **A+ (100%)** | **A (98%)** |
| 1. **There is insufficient high-level evidence to recommend the routine use of OMMs for weight loss before MBS** | **A+ (100%)** | **Agree** | **Agree** | **Agree** | **Agree** | **Agree** | **Agree** | **Agree** | **Agree** | **Disagree** | **Agree** | **Disagree** | **B (82%)** | **A (91%)** |
| 1. **The decision to use OMMs before MBS should be personalized to determine the most appropriate strategy for each patient’s circumstances** | **A+ (100%)** | **Agree** | **Agree** | **Agree** | **Agree** | **Agree** | **Agree** | **Agree** | **Agree** | **Agree** | **Agree** | **Agree** | **A+ (100%)** | **A+ (100%)** |
| 1. **Future research is needed to explore the value of using OMMs before MBS to assess their benefits, risks, and clinical outcomes** | **A+ (100%)** | **Agree** | **Agree** | **Agree** | **Agree** | **Agree** | **Agree** | **Agree** | **Agree** | **Agree** | **Agree** | **Agree** | **A+ (100%)** | **A+ (100%)** |
| 1. **Healthy nutrition, including adequate protein consumption, as well as resistance exercise, is recommended for those treated with OMMs before MBS** | **A (97%)** | **Agree** | **Agree** | **Agree** | **Agree** | **Agree** | **Agree** | **Agree** | **Agree** | **Agree** | **Agree** | **Agree** | **A+ (100%)** | **A (98%)** |
| 1. **In general, preoperative treatment with OMMs should be discontinued before MBS to minimize perioperative risk** | **A (94%)** | **Agree** | **Agree** | **Agree** | **Agree** | **Agree** | **Agree** | **Agree** | **Agree** | **Agree** | **Agree** | **Agree** | **A+ (100%)** | **A (97%)** |
| 1. **Treatments with OMMs after MBS should generally be withheld until the achievement of the weight plateau, unless there is a compelling clinical need for earlier initiation** | **A+ (100%)** | **Agree** | **Agree** | **Agree** | **Agree** | **Agree** | **Agree** | **Agree** | **Agree** | **Agree** | **Agree** | **Agree** | **A+ (100%)** | **A+ (100%)** |
| 1. **Future research is needed to identify predictors of which patients are likely to derive substantial benefit from combined pharmaco-surgical therapy for obesity and its complications** | **A+ (100%)** | **Agree** | **Agree** | **Agree** | **Agree** | **Agree** | **Agree** | **Agree** | **Agree** | **Agree** | **Agree** | **Agree** | **A+ (100%)** | **A+ (100%)** |
| 1. **MBS is strongly associated with reduced adverse cardiovascular events, and GLP1RA agonists have been shown to reduce such events. Future research is required to determine the benefits of combination treatment for these outcomes.** | **A+ (100%)** | **Agree** | **Agree** | **Agree** | **Agree** | **Agree** | **Agree** | **Agree** | **Agree** | **Agree** | **Agree** | **Agree** | **A+ (100%)** | **A+ (100%)** |
| 1. **Both MBS and GLP1RA agonists reduce chronic kidney disease. Future research is required to determine the benefits of combination treatment for these outcomes.** | **A+ (100%)** | **Agree** | **Agree** | **Agree** | **Agree** | **Agree** | **Agree** | **Agree** | **Agree** | **Agree** | **Agree** | **Agree** | **A+ (100%)** | **A+ (100%)** |
| 1. **In patients with a suboptimal clinical response after MBS, the addition of OMMs can improve metabolic outcomes** | **A+ (100%)** | **Agree** | **Agree** | **Agree** | **Agree** | **Agree** | **Agree** | **Agree** | **Agree** | **Agree** | **Agree** | **Agree** | **A+ (100%)** | **A+ (100%)** |
| 1. **For patients requiring OMMs to maintain a healthy weight after MBS, the ongoing use of the medications is likely needed** | **A (94%)** | **Agree** | **Agree** | **Agree** | **Agree** | **Agree** | **Agree** | **Agree** | **Agree** | **Agree** | **Agree** | **Agree** | **A+ (100%)** | **A (97%)** |
| 1. **Research on the intermittent use of OMMs and/or their dose adjustment after MBS with a suboptimal response is needed** | **A (94%)** | **Agree** | **Agree** | **Agree** | **Agree** | **Agree** | **Agree** | **Agree** | **Agree** | **Agree** | **Agree** | **Agree** | **A+ (100%)** | **A (97%)** |
| 1. **The benefit of endoscopic therapies for obesity can be enhanced by the combination with OMMs** | **C (74%)** | **Agree** | **Agree** | **Agree** | **Agree** | **Agree** | **Agree** | **Agree** | **Agree** | **Agree** | **Agree** | **Agree** | **A+ (100%)** | **B (87%)** |
| 1. **Patients with a suboptimal initial response or recurrent weight gain after MBS should be informed of all available evidence-based treatments, including their benefits and risks** | **A+ (100%)** | **Agree** | **Agree** | **Agree** | **Agree** | **Agree** | **Agree** | **Agree** | **Agree** | **Agree** | **Agree** | **Agree** | **A+ (100%)** | **A+ (100%)** |
| 1. **In patients with a suboptimal initial response or recurrent weight gain after MBS, different options, including OMMs, endoscopic therapies, and revisional and conversion surgery, can be considered** | **A (94%)** | **Agree** | **Agree** | **Agree** | **Agree** | **Agree** | **Agree** | **Agree** | **Agree** | **Agree** | **Agree** | **Agree** | **A+ (100%)** | **A (97%)** |
| 1. **Emerging evidence indicates that the weight loss induced by OMMs is similar among people who have or have not undergone MBS** | **A+ (100%)** | **Agree** | **Agree** | **Agree** | **Agree** | **Agree** | **Agree** | **Agree** | **Agree** | **Agree** | **Agree** | **Agree** | **A+ (100%)** | **A+ (100%)** |
| 1. **When used after MBS, there appears to be no increased incidence of side effects of OMMs compared to non-surgical cohorts** | **A (97%)** | **Agree** | **Agree** | **Agree** | **Agree** | **Agree** | **Agree** | **Agree** | **Agree** | **Agree** | **Agree** | **Agree** | **A+ (100%)** | **A (98%)** |
| 1. **As the long-term efficacy and safety of OMMs after MBS is unknown, studies are needed to understand the value and limitations of such combination therapy** | **A+ (100%)** | **Agree** | **Agree** | **Agree** | **Agree** | **Agree** | **Agree** | **Agree** | **Agree** | **Agree** | **Agree** | **Agree** | **A+ (100%)** | **A+ (100%)** |
| 1. **Endpoints of future clinical trials of existing and/or novel obesity-management interventions (behavioral, pharmacological, endoscopic, and surgical) should focus on improvement, remission, and prevention of clinical manifestations and complications of obesity in addition to weight loss** | **A+ (100%)** | **Agree** | **Agree** | **Agree** | **Agree** | **Agree** | **Agree** | **Agree** | **Agree** | **Agree** | **Agree** | **Agree** | **A+ (100%)** | **A+ (100%)** |
| 1. **Studies are needed to define stage-specific therapeutic protocols that integrate surgical intervention and adjuvant pharmacotherapy to achieve improvement (or remission when possible) of clinical obesity** | **A (95%)** | **Agree** | **Agree** | **Agree** | **Agree** | **Agree** | **Agree** | **Agree** | **Agree** | **Agree** | **Agree** | **Agree** | **A+ (100%)** | **A (97%)** |
| 1. **Further investigation of the mechanisms of action of distinct MBS procedures is an important research priority to understand the additive vs. synergistic effects of different possible combinations of surgical and drug-based therapies. This knowledge is necessary to optimize the safety and efficacy of adjuvant pharmacotherapy for obesity.** | **A (95%)** | **Agree** | **Agree** | **Agree** | **Agree** | **Agree** | **Agree** | **Agree** | **Agree** | **Agree** | **Agree** | **Agree** | **A+ (100%)** | **A (97%)** |
| 1. **For patients with recurrent weight gain, treatment with available OMMs should be considered prior to revisional surgery.** | **A (92%)** | **Agree** | **Agree** | **Agree** | **Agree** | **Agree** | **Agree** | **Agree** | **Agree** | **Agree** | **Agree** | **Agree** | **A+ (100%)** | **A (96%)** |
| 1. **If treatment with OMMs after MBS results in a suboptimal clinical response or if there is an inability to continue medications (e.g., due to cost or an adverse reaction), then endoscopic, revision, or conversion surgery should be considered.** | **A+ (100%)** | **Agree** | **Agree** | **Agree** | **Agree** | **Agree** | **Agree** | **Agree** | **Agree** | **Agree** | **Agree** | **Agree** | **A+ (100%)** | **A+ (100%)** |
| 1. **People living with obesity need access to all evidence-based treatments, including MBS and OMMs, as part of standard healthcare services** | **A (95%)** | **Agree** | **Agree** | **Agree** | **Agree** | **Agree** | **Agree** | **Agree** | **Agree** | **Agree** | **Agree** | **Agree** | **A+ (100%)** | **A (97%)** |
| 1. **Health systems need to support the long-term management of obesity as they do for other chronic diseases (e.g., diabetes or cardiovascular disease)** | **A (95%)** | **Agree** | **Agree** | **Agree** | **Agree** | **Agree** | **Agree** | **Agree** | **Agree** | **Agree** | **Agree** | **Agree** | **A+ (100%)** | **A (97%)** |
| 1. **All healthcare providers need a basic understanding of the complex etiology, pathophysiology, and evidence-based management of obesity** | **A+ (100%)** | **Agree** | **Agree** | **Agree** | **Agree** | **Agree** | **Agree** | **Agree** | **Agree** | **Agree** | **Agree** | **Agree** | **A+ (100%)** | **A+ (100%)** |
| 1. **Studies on the cost-effectiveness of the association of modern pharmacotherapy and MBS are essential to determine the role of preoperative and postoperative OMMs** | **A+ (100%)** | **Agree** | **Agree** | **Agree** | **Agree** | **Agree** | **Agree** | **Agree** | **Agree** | **Agree** | **Agree** | **Agree** | **A+ (100%)** | **A+ (100%)** |
| 1. **Similar benefit-risk and benefit-cost considerations, and therefore willingness to pay, should be applied to the treatment of obesity as they are to other chronic diseases** | **A+ (100%)** | **Agree** | **Agree** | **Agree** | **Agree** | **Agree** | **Agree** | **Agree** | **Agree** | **Agree** | **Agree** | **Agree** | **A+ (100%)** | **A+ (100%)** |

**100% consensus support were considered grade A+, statements that received 90–99.9% consensus support were considered grade A, statements that received 80–89.9% consensus support were considered grade B, statements that received 70–79.9% consensus support were considered grade C, and statements that received 66–69.9% consensus support were considered grade D. Statements that received less than 66% consensus support were considered to have failed consensus**
